# Supplementary material for: DNA methylation around transcription start sites is not globally associated with transcription in the grain of natural and synthetic hexaploid wheat
Source: BMC Plant Biol. 2026 Mar 20;26:767. doi: 10.1186/s12870-026-08607-6 (PMC13126883; doi:10.1186/s12870-026-08607-6)
Supplement: Supplementary file 2 — Additional file 2. Supplementary Figures 1-12 (.pdf format). [file 12870_2026_8607_MOESM2_ESM.pdf]

DNA methylation around transcription start sites not globally associated with transcription in the grain of natural and synthetic hexaploid wheat

Meriem Banouh, Mamadou Dia Sow, Caroline Pont, Michael A. Seidel, Jerome Salse, Peter Civan

Supplementary Figures 1-12

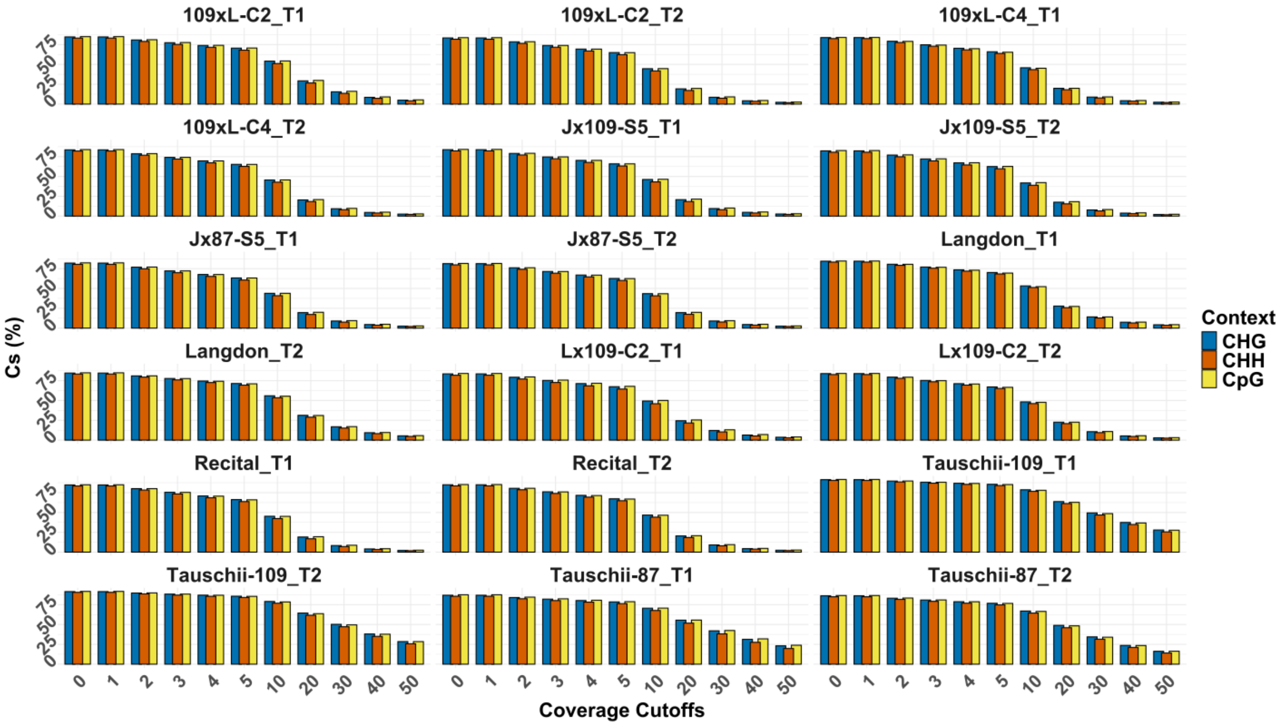

Supplementary Fig. 1. Coverage of target cytosine sites at various depth thresholds. Percentage of target cytosines covered by data at depth thresholds indicated on the x-axis. Each library was processed separately, with suffixes T1 and T2 indicating the biological replicates.

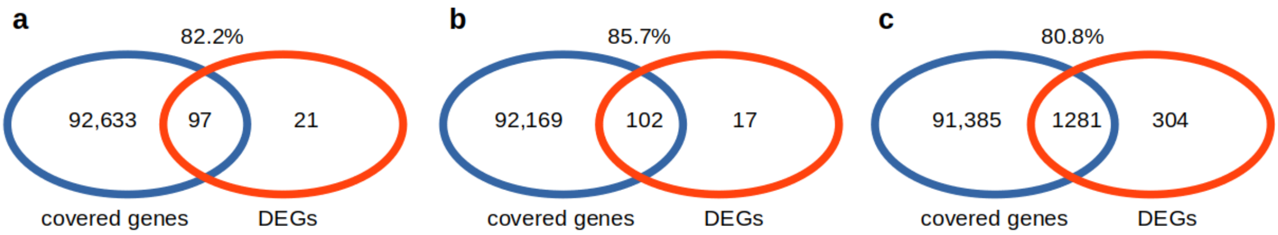

Supplementary Fig. 2. Coverage of DEGs identified in Banouh et al. (2023). Total number of high-confidence genes (Chinese Spring v1.1 annotation) covered at the 3x depth threshold, in relation to DEGs identified previously from the same samples and analyzed in this paper. (a) 109xL-C2; (b) 109xL-C4; (c) Lx109-C2.

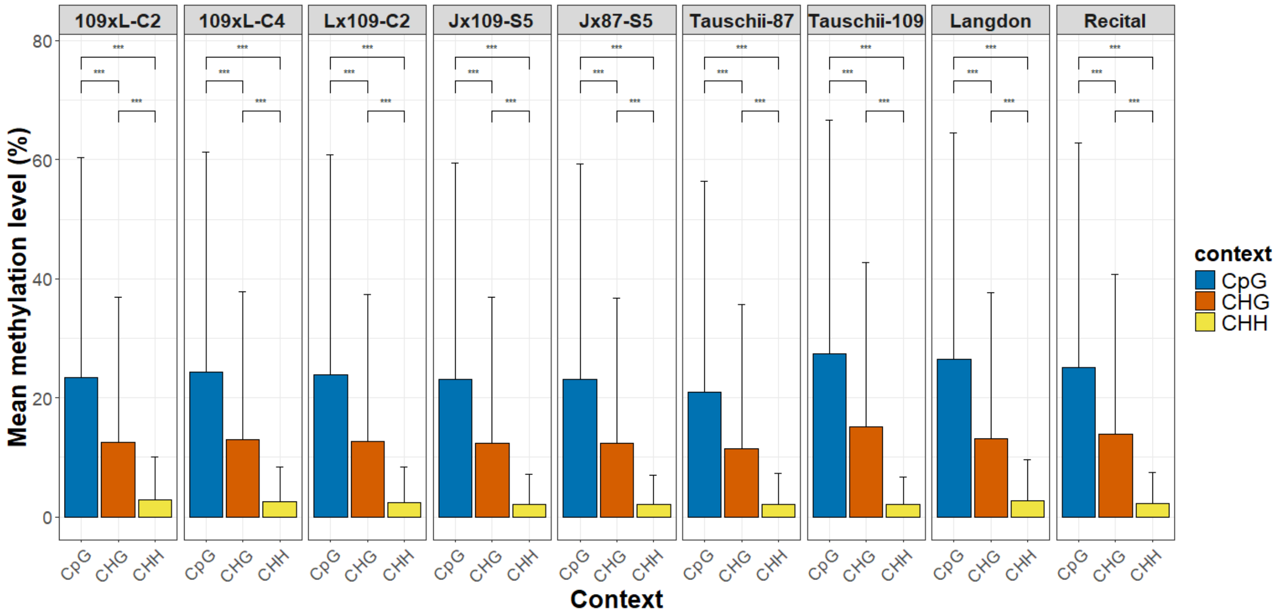

Supplementary Fig. 3. Mean methylation levels per sequence context (CpG, CHG, CHH) across different genotypes. All sites passing the 10x coverage in a genotype were included and were not filtered for consistency across genotypes (e.g., positions analyzed in Tauschii-87 and Tauschii-109 have only a partial overlap). Methylation levels are therefore compared only across contexts within genotypes, and not across genotypes. Statistical significance (Wilcoxon test) is indicated with asterisks (*** $p < 0.001$).

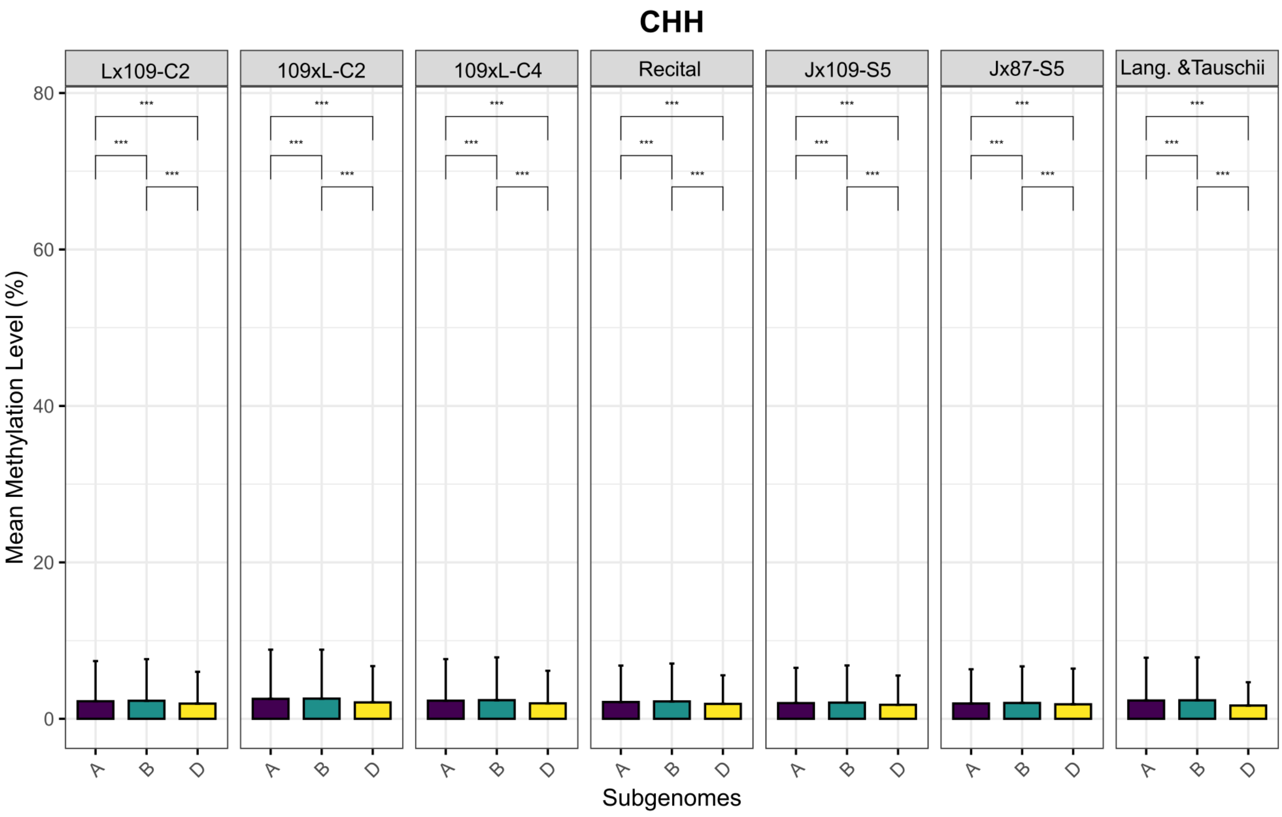

Supplementary Fig. 4. Mean Methylation Levels per subgenome (A, B and D) across genotypes in CHH contexts (*** $p < 0.001$). Error bars represent the standard deviation.

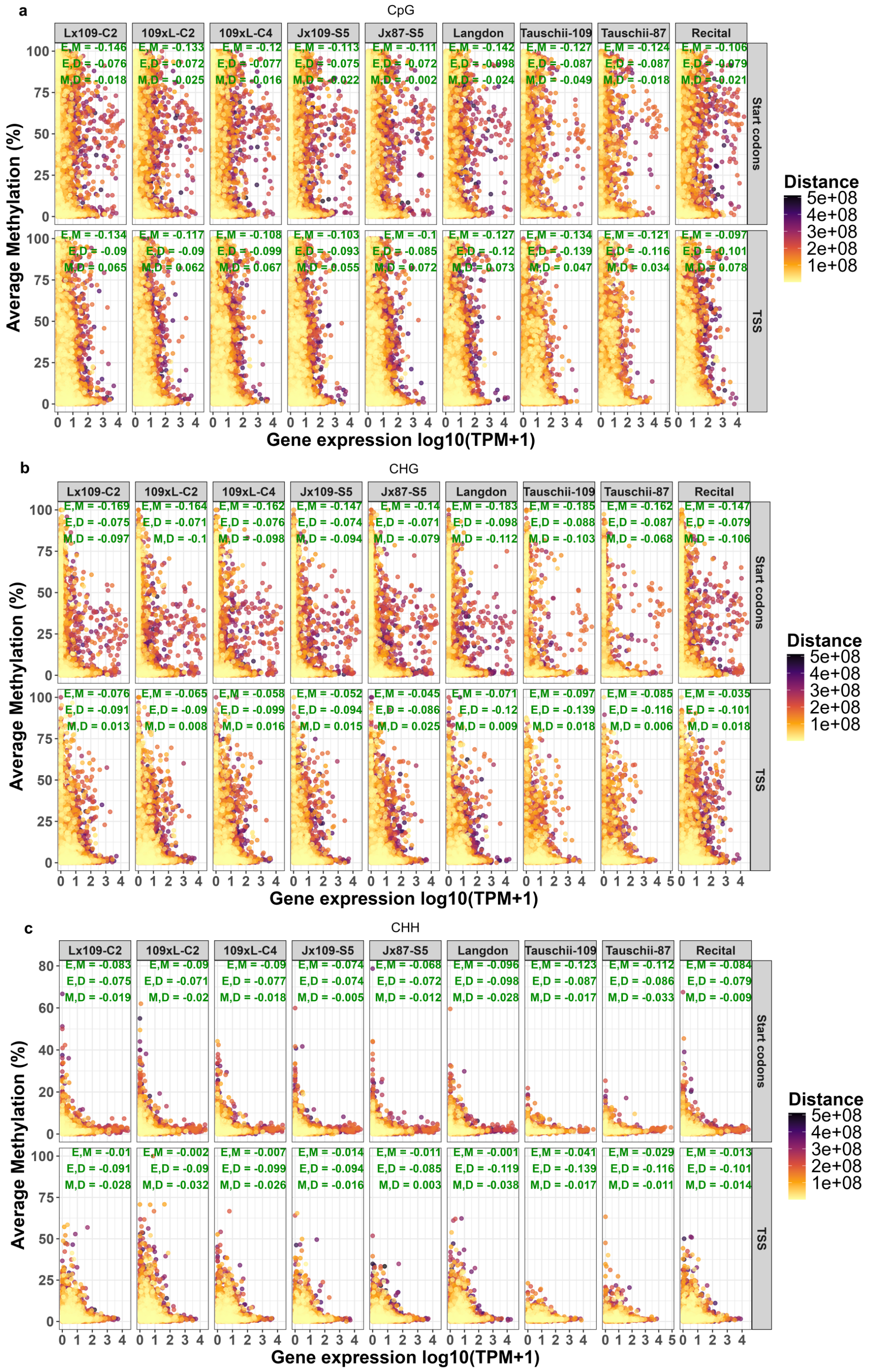

Supplementary Fig. 5. The relationship between transcription (x-axis), DNA methylation level (y-axis), and centromere distance (expressed with a color code). Pearson's correlation between gene expression and DNA methylation (E,M), gene expression and centromere distance (E,D), and DNA methylation and centromere distance (M,D) is show in green for each scatter plot. DNA methylation is shown separately for the CpG context (a), CHG context (b) and CHH context (c). For each context, the top panels show the SC-centered data set, while the bottom panels show TSS-centered data set.

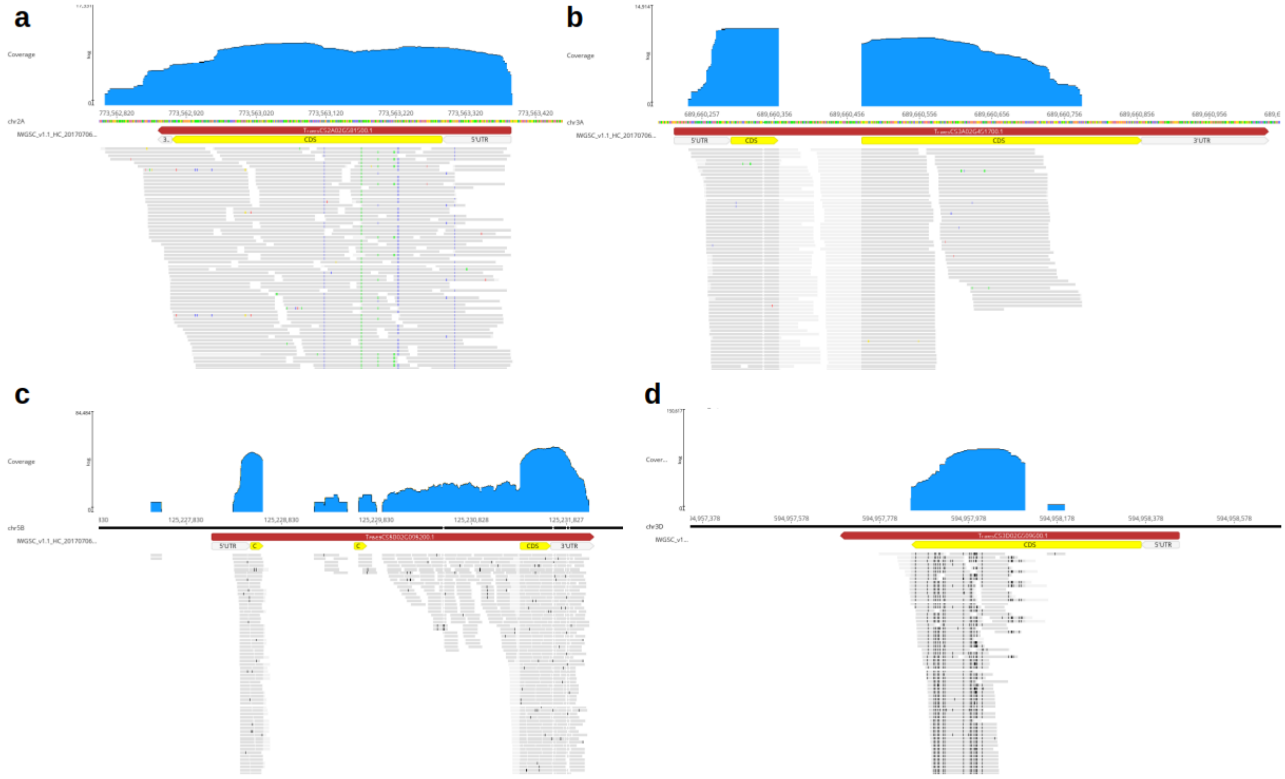

Supplementary Fig. 6. Examples of 5'UTR annotation in genes classified as highly-expressed with highly methylated TSS. The images show RNA-seq reads aligned to the IWGSC v1.1-annotated reference, visualized in Geneious 6.1 (https://www.geneious.com). Genes are indicated with red tracks; coding sequences as yellow tracks, and UTRs as light-gray tracks. In our analyses, the first position of the 5'UTRs was considered as TSS. Reads are shown as grey, horizontal bars, with their density indicated on a logarithmic scale above the reference as areas in blue. (a) A gene where most transcription starts exactly at the beginning of the annotated 5'UTR. (b) a gene where most transcription starts ~75 bp downstream of the inferred TSS, but still within the region targeted by the capture design and analyzed (-150 bp and +150 bp, relative to the inferred TSS). (c) A gene where most transcription starts ~300 bp downstream of the inferred TSS. (d) A gene where the real TSS is unclear, apparently due to alignment problems (intron-exon structure; sequence divergence between the sample and the reference).

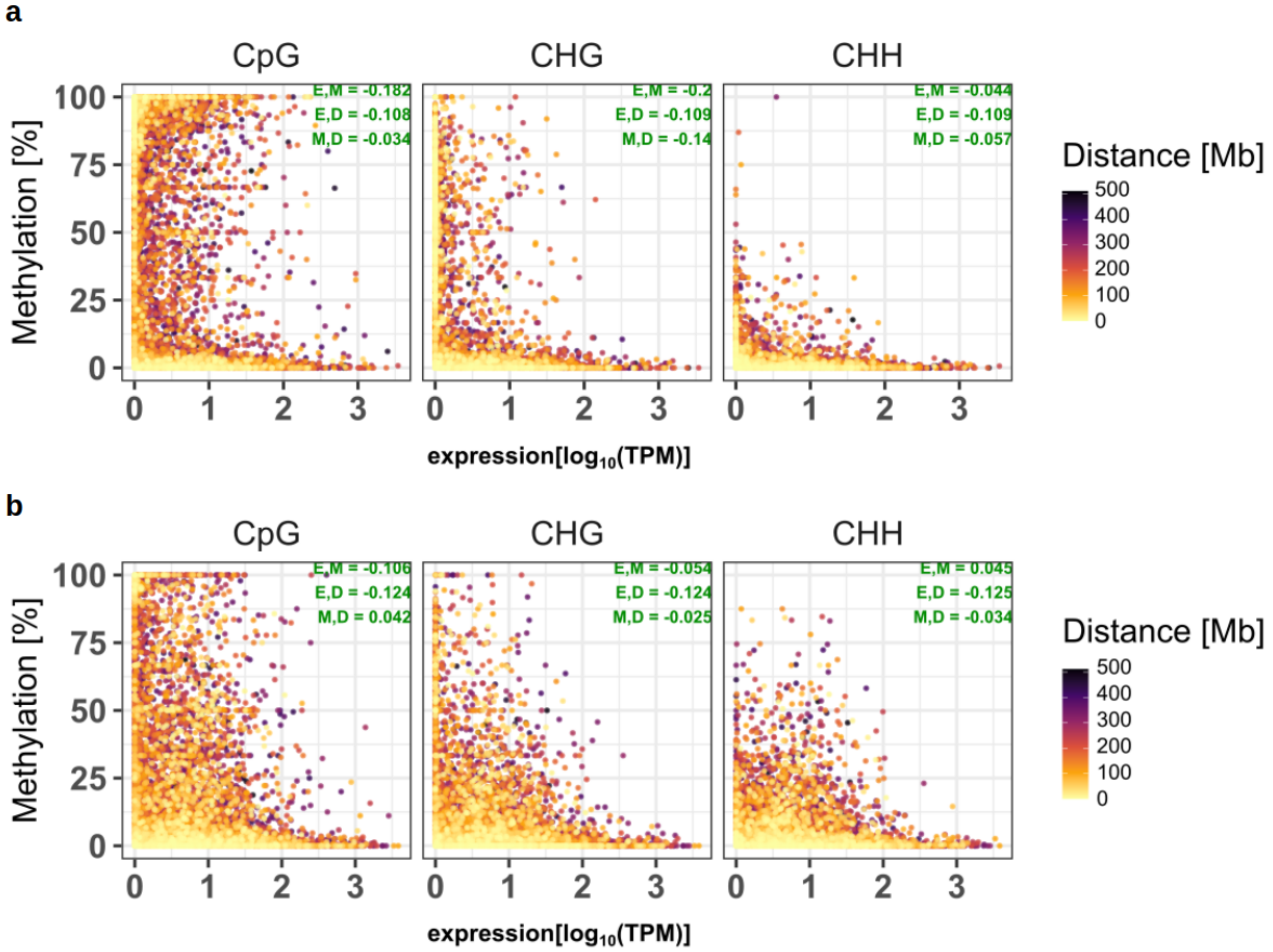

Supplementary Fig. 7. The relationship between transcription (x-axis), DNA methylation level (y-axis), and centromere distance (expressed with a color code) in two-week-old leaves of cv. Chinese Spring (public datasets SRP133674 and SRP133837). Pearson's correlation between gene expression and DNA methylation (E,M), gene expression and centromere distance (E,D), and DNA methylation and centromere distance (M,D) is show in green for each scatter plot. (a) The subset of genes with the SC methylation data. (b) The subset of genes with the TSS methylation data.

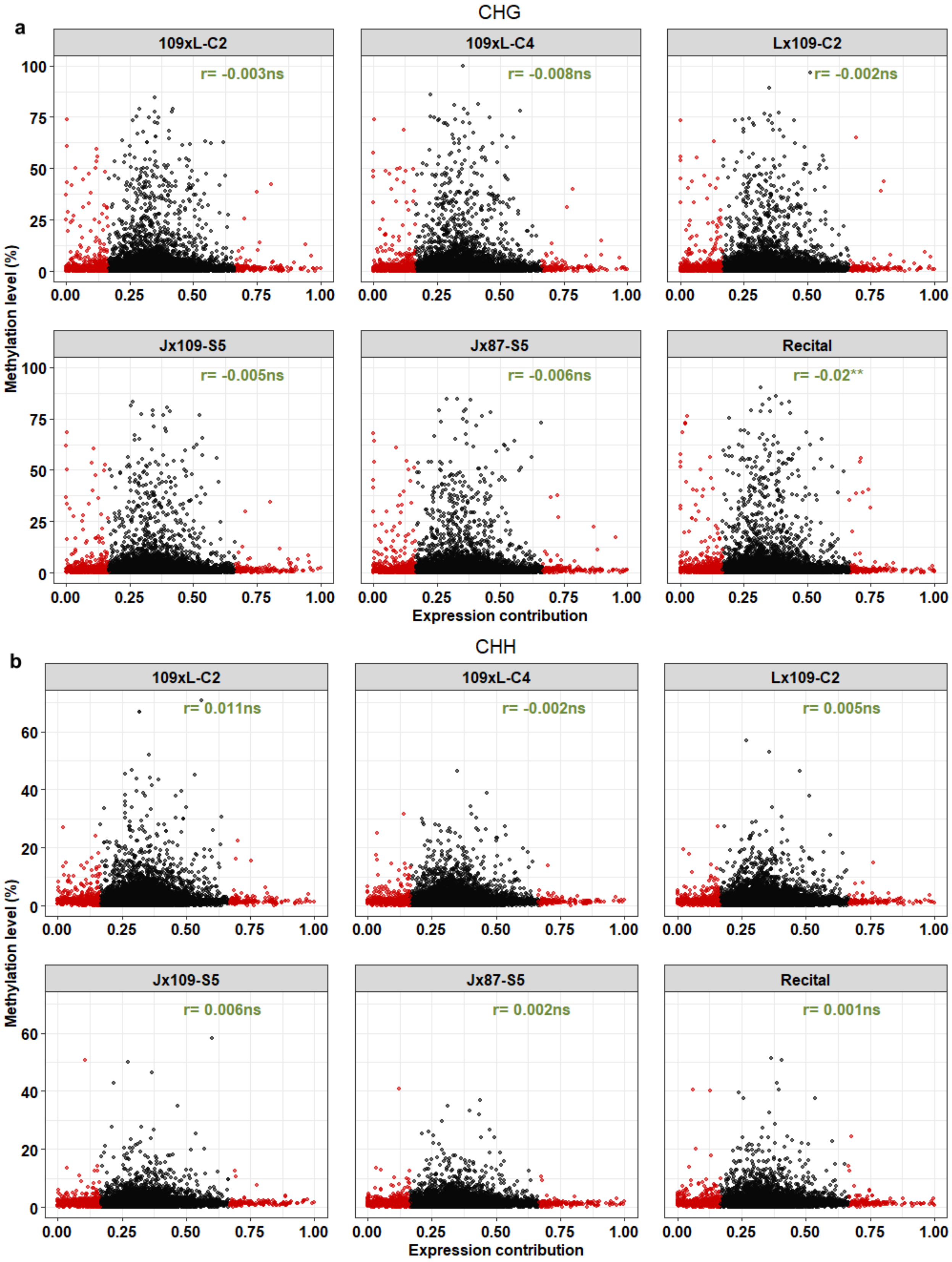

Supplementary Fig. 8. Scatter plots showing the relationship between methylation level and expression contribution to the triad. Suppressed and dominant genes are shown in red. (a) CHG context (b) CHH contexts. Pearson's correlation coefficient are shown for each sample in green (ns, $p > 0.05$; ** $p < 0.01$).

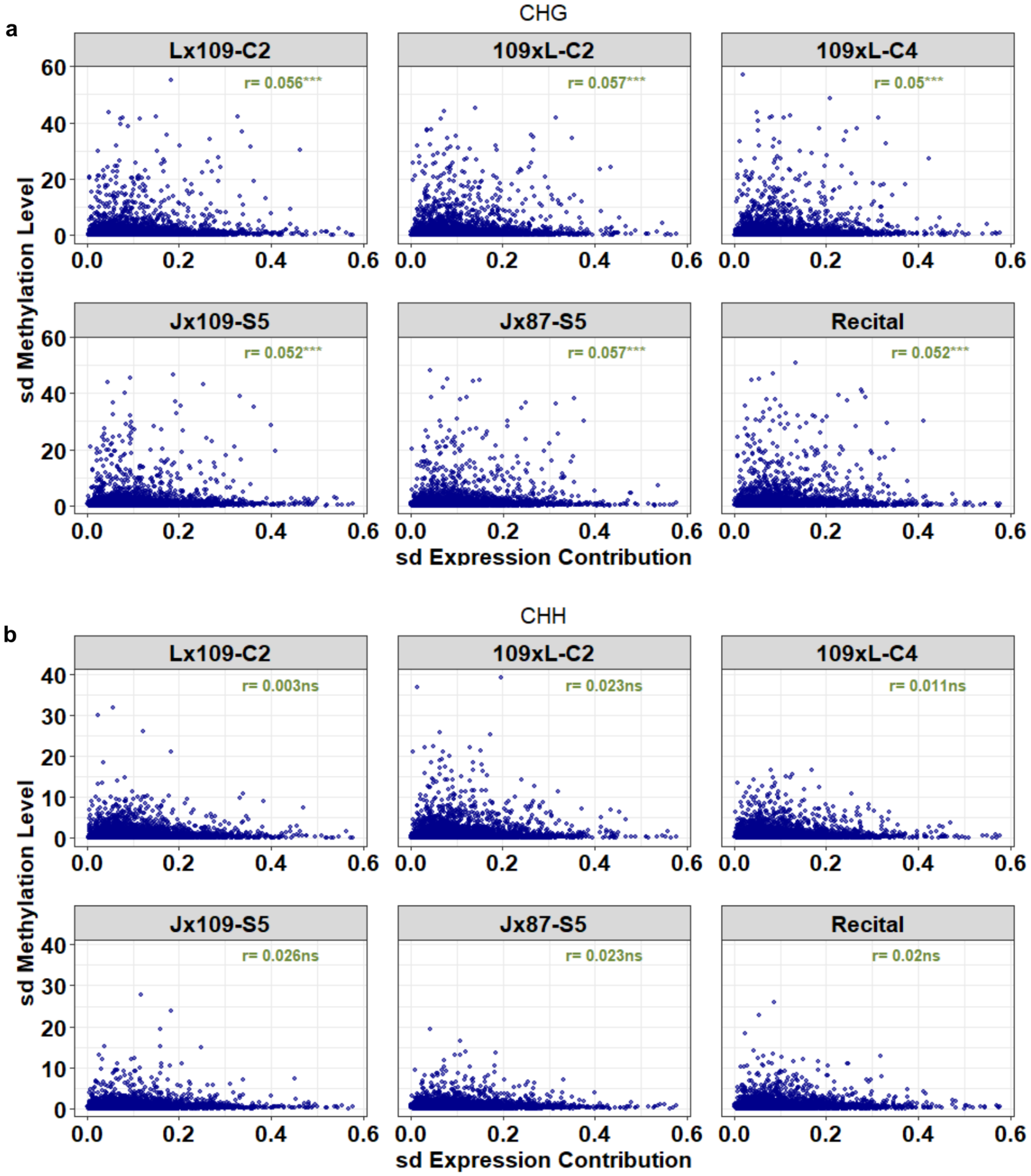

Supplementary Fig. 9. Scatter plots showing the correlation between the variance (sd) in expression contribution within triads, and the corresponding variance in methylation levels within triads in (a) CHG and (b) CHH contexts. Pearson's correlation coefficients are shown for each sample in green (ns, p >0.05; *** $p < 0.001$).

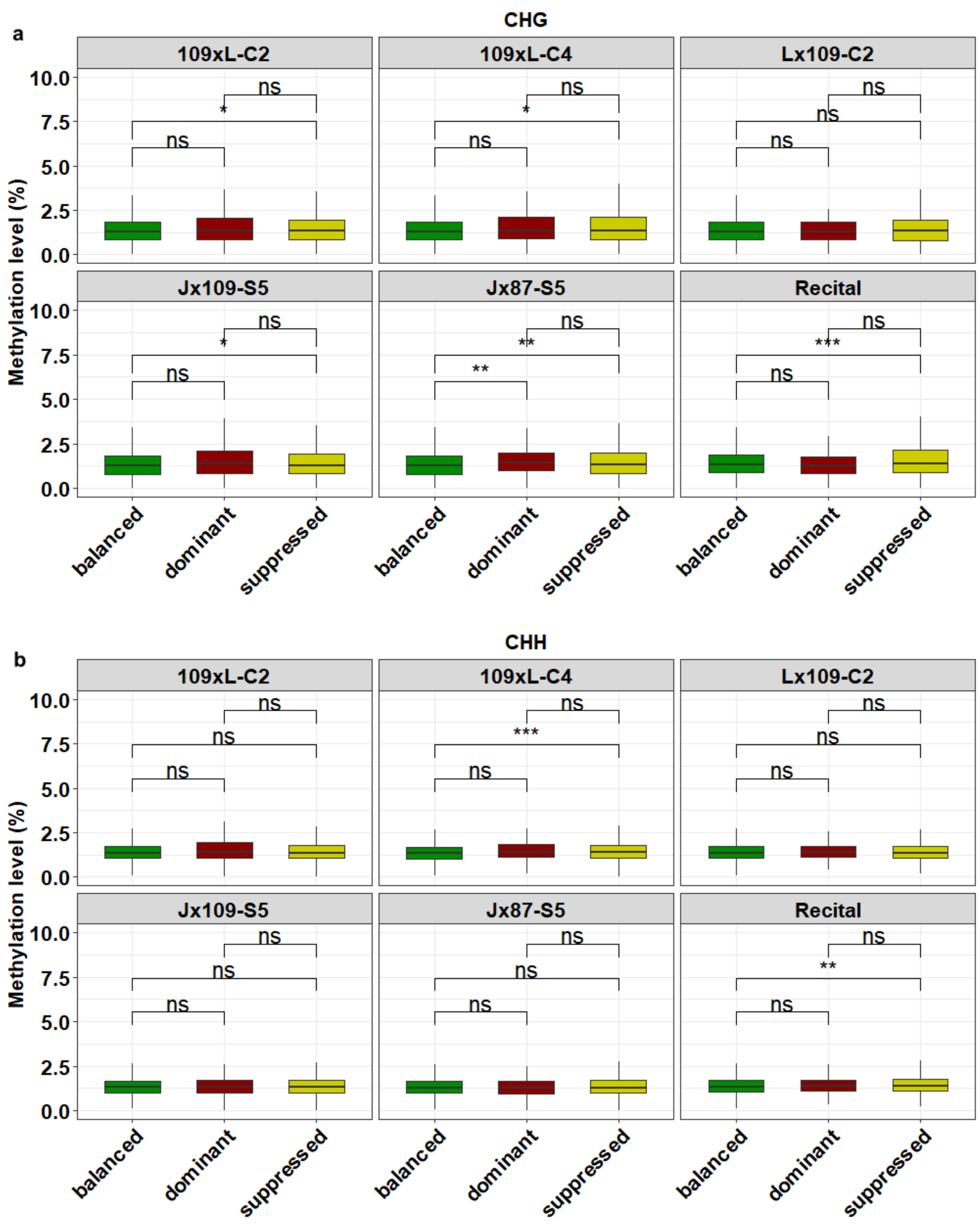

Supplementary Fig. 10. Comparisons of mean methylation between balanced, suppressed and dominant genes across samples with error bars representing the mean ± SD. Asterisks indicate significant differences based on the Wilcoxon test (ns = not significant, * $p < 0.05$, ** $p < 0.01$, *** $p < 0.001$).

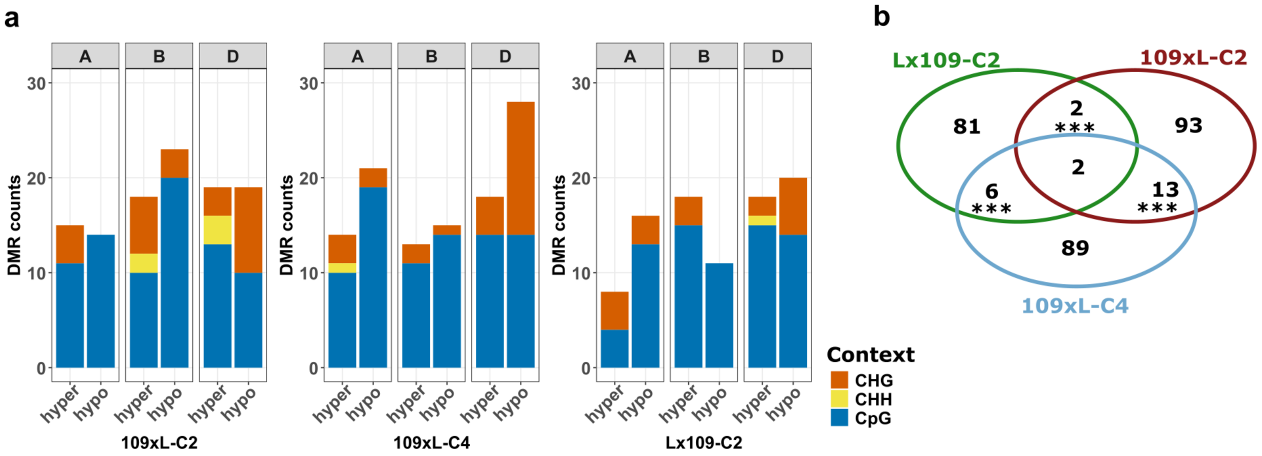

Supplementary Fig. 11. DMRs identification using 300bp windows as regions. (a) Counts of hyper/hypomethylated DMRs in different hexaploid synthetics across different subgenomes and different cytosine contexts. (b) Venn diagram showing the overlap of DMRs (considering all contexts) detected in different synthetic samples. Asterisks indicate the significance of the overlaps (*** $p < 0.001$).

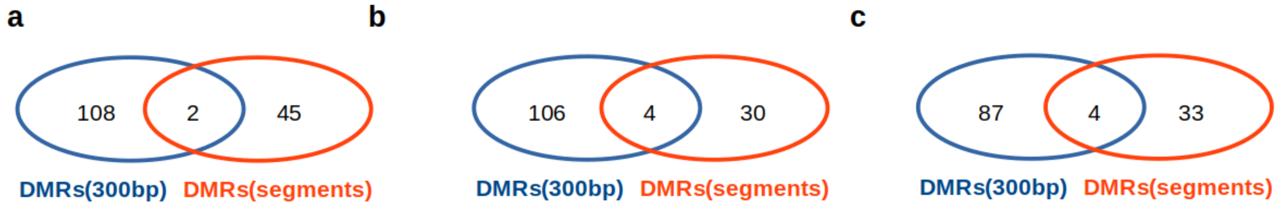

Supplementary Fig. 12. Venn diagrams showing the concordance (or lack thereof) of the two methods used for the DMR detection (considering all contexts). (a) 109xL-C2; (b) 109xL-C4; (c) Lx109-C2.
